# Plasma microRNA Levels Combined with CEA and CA19-9 in the Follow-Up of Colorectal Cancer Patients

**DOI:** 10.3390/cancers11060864

**Published:** 2019-06-21

**Authors:** Martin Pesta, Radek Kucera, Ondrej Topolcan, Marie Karlikova, Katerina Houfkova, Jiri Polivka, Tereza Macanova, Iva Machova, David Slouka, Vlastimil Kulda

**Affiliations:** 1 Department of Biology, Faculty of Medicine in Pilsen, Charles University, alej Svobody 76, 32300 Pilsen, Czech Republic; martin.pesta@lfp.cuni.cz (M.P.); katerina.houfkova@lfp.cuni.cz (K.H.); tereza.macanova@lfp.cuni.cz (T.M.); 2 Laboratory of Immunoanalysis, University Hospital in Pilsen, E. Benese 13, 30599 Pilsen, Czech Republic; kucerar@fnplzen.cz (R.K.); topolcan@fnplzen.cz (O.T.); karlikovam@fnplzen.cz (M.K.); jiri.polivka@lfp.cuni.cz (J.P.); iva.machova@lfp.cuni.cz (I.M.); slouka@fnplzen.cz (D.S.); 3 Department of Medical Chemistry and Biochemistry, Faculty of Medicine in Pilsen, Charles University, Karlovarska 48, 30166 Pilsen, Czech Republic; 4 Department of Histology and Embryology, Faculty of Medicine in Pilsen, Charles University, Karlovarska 48, 30166 Pilsen, Czech Republic

**Keywords:** colorectal cancer, biomarkers, recurrence, microRNA, CEA, CA19-9, blood plasma

## Abstract

Colorectal cancer (CRC) ranks among the most common cancers worldwide. Surgical removal remains the best strategy for treatment of resectable tumors. An important part of caring for patients after surgery is monitoring for early detection of a possible relapse of the disease. Efforts are being made to improve the sensitivity and specificity of routinely used carcinoembryonic antigen (CEA) with the use of additional biomarkers such as microRNAs. The aim of our study was to evaluate the prognostic potential of microRNAs and their use as markers of disease recurrence. The quantitative estimation of CEA, CA19-9, and 22 selected microRNAs (TaqMan Advanced miRNA Assays) was performed in 85 paired (preoperative and postoperative) blood plasma samples of CRC patients and in samples taken during the follow-up period. We have revealed a statistically significant decrease in plasma levels for miR-20a, miR-23a, miR-210, and miR-223a (*p* = 0.0093, *p* = 0.0013, *p* = 0.0392, and *p* = 0.0214, respectively) after surgical removal of the tumor tissue. A statistically significant relation to prognosis (overall survival; OS) was recorded for preoperative plasma levels of miR-20a, miR-21, and miR-23a (*p* = 0.0236, *p* = 0.0316, and *p* =0.0271, respectively) in a subgroup of patients who underwent palliative surgery. The best discrimination between patients with favorable and unfavorable outcomes was achieved by a combination of CEA, CA19-9 with miR-21, miR-20a, and miR-23a (*p* < 0.0001). The use of these microRNAs for early disease recurrence detection was affected by a low specificity in comparison with CEA and CA19-9. CEA and CA19-9 had high specificity but low sensitivity. Our results show the benefit of combining currently used standard biomarkers and microRNAs for precise prognosis estimation.

## 1. Introduction

Colorectal cancer (CRC) ranks among the most common cancers worldwide [1]. In the last 20 years, we have witnessed the enlargement of the spectrum of available treatment modalities, especially in the fields of chemotherapy, targeted therapy, and immunotherapy [2,3]. Nevertheless, early surgical removal remains the best strategy for the treatment of resectable tumors. In addition to biomarkers for early disease detection (screening) and prediction of treatment response, there is also a need for efficient biomarkers for early detection of disease recurrence in the follow-up period of patients.

Approximately 20% of surgically treated CRC patients will suffer from disease recurrence within five years after surgery [4]. For the purpose of early detection of recurrence, the preferred examination must be minimally invasive and repeatable over time. Therefore, the most suitable biological material is peripheral venous blood. Carcinoembryonic antigen (CEA) is a well-established marker for this purpose, recommended by both the American Society of Clinical Oncology (ASCO) and the European Group on Tumor Markers (EGTM) [5,6]. There is good evidence that routine CEA monitoring during the postresection follow-up period detects metastatic disease. However, the sensitivity of this marker is not considered to be sufficient [7]. The sensitivity of CEA is compromised by the lack of consistency in the increase in its plasma levels. There are CRC patients with poorly differentiated tumors with CEA only present at low concentrations or even at undetectable levels [8].

There are efforts to improve the sensitivity and specificity by using additional tumor markers like cancer antigen 19-9 (CA19-9) [9], but also using biomarkers reflecting other characteristics of tumor cells. Just such a group of molecules with new and promising features are microRNAs (miRNAs) [10]. MiRNAs are small, non-coding RNA molecules that play a complex role in the post-transcriptional regulation of gene expression, including ceRNA regulatory networks. The human genome encodes over 2500 miRNAs [11], which may target about 60% of mammalian genes [12]. Many studies have described changes in the expression of miRNAs and their involvement in carcinogenesis, tumor progression, development of metastases, and their relation to prognosis and the effects of treatment in many types of cancer [13,14,15], including CRC [16,17]. Gene expression profiling has revealed patterns of miRNA expression characteristic for these processes [18].

The key role of miRNAs is in the regulation of cellular processes including those involved in tumor growth. MiRNAs lack a direct executive function as enzymes, signaling molecules, or signal transduction molecules that are the targets of current anti-cancer therapy.

MiRNAs are released from cells into body fluids, and it is possible to detect them in blood plasma/serum as cell-free miRNAs or as cargo in exosomes as exosomal miRNAs.

From the point of view of analytical features, miRNA molecules exhibit high stability and can be easily assessed. However, despite the promising results of published studies on circulating miRNAs [19,20,21,22] used as biomarkers in CRC, no miRNA has yet proved robust enough to enter routine clinical use [23]. Therefore, the combining of already validated tumor markers (e.g., CEA, CA19-9) with new promising markers could be a way to make prognosis estimation and indication of disease recurrence more accurate.

Based on research of literature in the databases of PubMed and ISI Web of knowledge (published until February 2017) using the keywords “CRC” and “microRNA” and (“plasma” or “serum” or “circulating”), we selected 22 candidate miRNAs that have a potential role in CRC carcinogenesis. These miRNAs and references to studies based on which the selection was made are listed in the section Materials and Methods (Table 4).

The aim of the study was to identify miRNAs with a significant difference between preoperative and postoperative plasma levels, and to evaluate their ability to contribute to early detection of disease recurrence. We also evaluated the relation of these biomarkers to prognosis.

## 2. Results

First, we describe the changes revealed between the preoperative and postoperative plasma levels of the evaluated markers. We then show the relation of preoperative plasma levels of miRNAs to prognosis and the improvement of prognostic value achieved by an approach based on the combination of miRNAs and well-established, routinely used tumor markers (CEA, CA19-9). Finally, we demonstrate the use of these markers for the detection of disease recurrence.

From a set of 22 estimated miRNAs, we were able to obtain reliable values for only 9 (miR-20a-5p, miR-21-5p, miR-23a-3p, miR-29-3p, miR-92a-3p, miR-155-5p, miR-199a-3p, miR-210-3p, miR-223-3p). The others were excluded from evaluation due to high Ct values (Ct > 35) resulting in non-reliable quantification or no amplification.

### 2.1. Changes between Preoperative and Postoperative miRNA Plasma Levels

**Figure 1:**
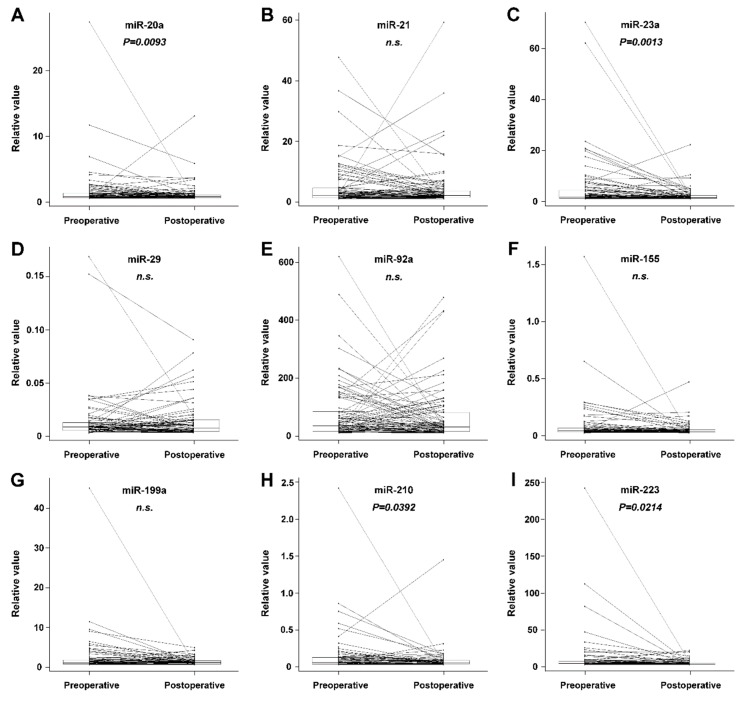
Comparison of paired preoperative and postoperative blood plasma miRNA values in the group comprising all patients. Box-and-whisker plots with outliers shown as separately plotted points. Paired values are connected by a line. A statistically significant decrease was recorded for miR-20a, miR-23a, miR-210, and miR-223. (n.s. = non-significant)

**Figure 2:**
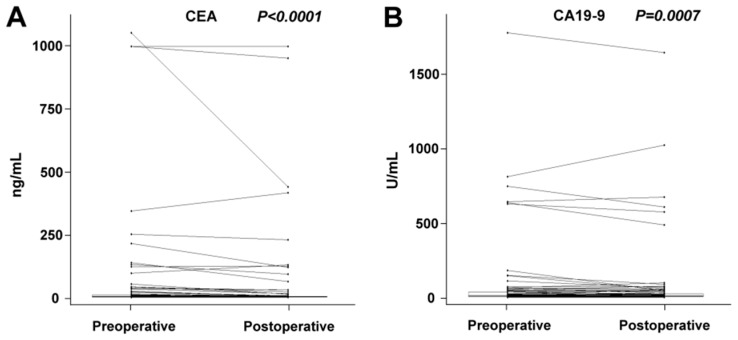
Comparison of paired preoperative and postoperative blood serum CEA (**A**) and CA19-9 (**B**) values in the group comprising all patients. Box-and-whisker plots with outliers shown as separately plotted points. Paired values are connected by a line. A statistically significant decrease was recorded for both CEA and CA19-9.

We have revealed a significant decrease in miR-21, miR-20a, miR-210, miR-23a, and miR-155 plasma levels after the complete removal of tumor mass (radical surgery). There was no significant change in miRNA plasma levels observed in the subgroup of patients who underwent palliative surgery. In the group comprising all patients (radical or palliative surgery), we revealed a statistically significant decrease in miR-20a, miR-23a, miR-210, and miR-223. The results on preoperative and postoperative changes in miRNA levels are summarized in Table 1. A postoperative decrease in plasma levels was also recorded for CEA and CA19-9 (Table 1). The comparison of preoperative and postoperative biomarker values in the group comprising all patients is shown graphically in Figure 1 and Figure 2. A dramatic decrease in postoperative levels of miRNAs is seen especially in patients with high preoperative levels.

### 2.2. The Relation of Preoperative Plasma Levels of miRNAs to Prognosis

**Figure 3:**
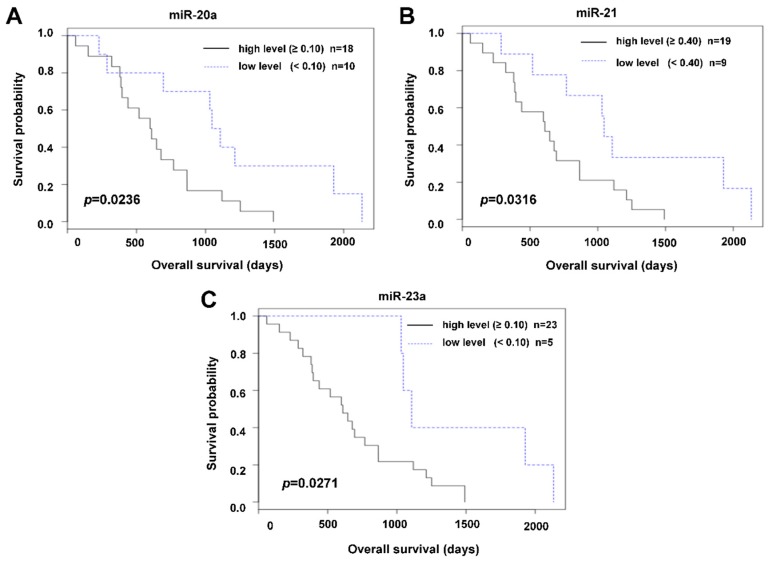
Relation of miR-20a (**A**), miR-21 (**B**), and miR-23a (**C**) preoperative plasma levels to overall survival (OS) in a group of patients who underwent palliative surgery (Kaplan–Meier curves).

The relation to prognosis (OS, overall survival) could only be evaluated in the subgroup of patients who underwent palliative surgery. Within the available follow-up, almost none of the patients who underwent radical surgery died, so no survival data were available. A statistically significant relation to OS was recorded for miR-21, miR-20a, and miR-23a (*p* = 0.0316, *p* = 0.0235, and *p* = 0.0270, respectively). The Kaplan–Meier survival distribution functions are shown in Figure 3.

**Figure 4:**
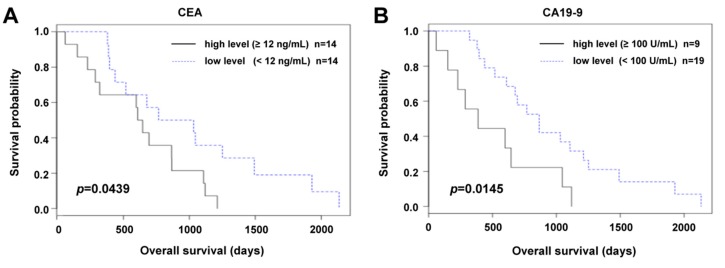
Relation of CEA (**A**) and CA19-9 (**B**) preoperative plasma levels to overall survival (OS) in a group of patients who underwent palliative surgery (Kaplan–Meier curves).

Likewise, we observed a statistically significant relation to OS of CEA and CA19-9 (*p* = 0.0439 and *p* = 0.0145, respectively); see Figure 4.

**Figure 5:**
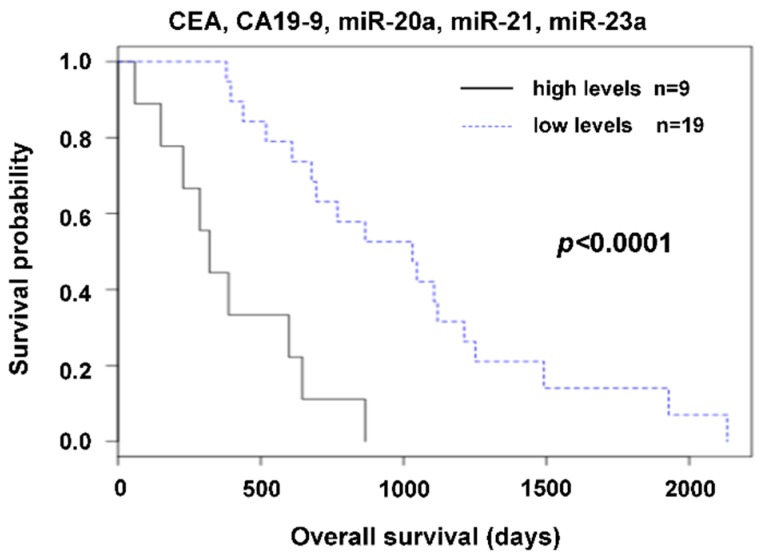
Combining CEA, CA19-9 with miR-20a, miR-21, and miR-23a. Patients with a very high value of any one of CEA, CA19-9, miR-20a, miR-21, or miR-23a had significantly shorter survival than those who had all the mentioned markers under the cut-off value.

The best discrimination between patients with favorable and unfavorable outcomes was achieved by the combination of estimated tumor markers with miRNAs. Patients with a very high value of any one of CEA, CA19-9, miR-20a, miR-21 or miR-23a, had significantly shorter survival than those who had all the mentioned markers under the cut-off value (Figure 5). It is important to mention that best discrimination between patients with a favorable and unfavorable prognosis is based on the inclusion of all those markers.

### 2.3. Detection of Disease Recurrence

**Figure 6:**
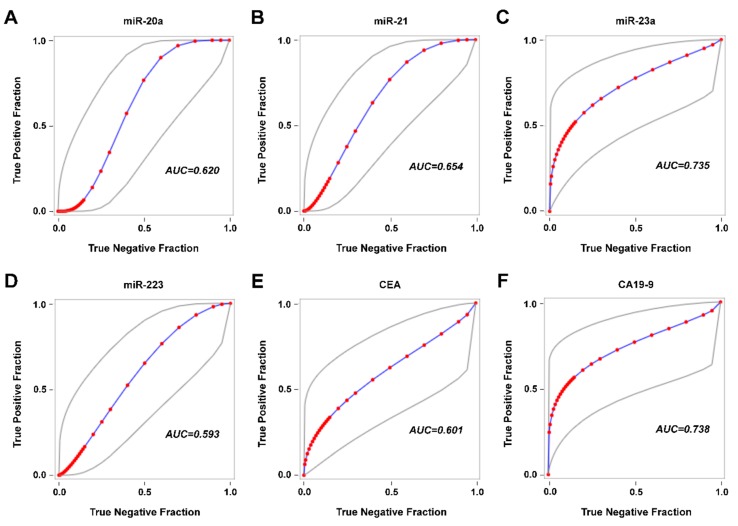
Receiver operating characteristic (ROC) curves for miR-20a (**A**), miR-21 (**B**), miR-23a (**C**), miR-223 (**D**), CEA (**E**), and CA19-9 (**F**). Abbreviations: AUC—area under the curve.

In the group of patients who underwent radical surgery, it was possible to estimate the sensitivity, specificity, positive predictive value, and negative predictive value of the markers for disease recurrence (Table 2). While CEA and CA19-9 had high specificity and low sensitivity, miRNAs suffered from false positives. Specificity of miRNAs was in the range of 50–60%. However, it is necessary to mention that in the case of follow-up, the data are limited by the small number of patients and the timing of sample collection. Receiver operating characteristic (ROC) curves for miR-20a, miR-21, miR-23a, miR-223, CEA, and CA19-9 are shown in Figure 6.

## 3. Discussion

Progress in the management of CRC patients has created a need for more accurate biomarkers to guide treatment and for early detection of disease recurrence. The biochemical gold standard for disease recurrence detection is CEA surveillance, and it is most effective when patients have high preoperative serum CEA levels. However, there are some limitations. Sensitivity is far from being sufficient [24]. Plasma concentration of CEA is not consistently elevated in colorectal cancer and may be undetectable or present at only low concentrations with poorly differentiated tumors [8]. There are efforts to improve the sensitivity and specificity using additional tumor markers like CA19-9 [9], but the impact on recurrence detection sensitivity improvement is still limited to those with elevated preoperative serum CA19-9 [25,26].

Progress in understanding the important role of some miRNAs in CRC pathogenesis and the promising features of these molecules from an analytical point of view (stability in body fluids) has led to the possibility of using these molecules for CRC surveillance. miRNAs selected for this study have been previously described as those related to CRC carcinogenesis and could be candidate CRC biomarkers (Table 4). Their plasma levels are expected to be related to the actual process of pathogenesis. This study has revealed miRNAs (miR-20a, miR-210, miR-223, and miR-23a) with a significant difference between preoperative and postoperative plasma levels in a group of surgically treated CRC patients. Detailed data are shown in Table 1 and Figure 1. The study group consisted of two categories of patients: those who underwent radical or palliative surgery. In radically treated patients, it can be assumed that the complete removal of tumor mass will result in a decrease in miRNA levels, while palliative intervention does not lead to the complete removal of tumor mass, so a decrease in miRNA levels may not be significant.

Nevertheless, preoperative plasma values are independent of intervention type, be it radical or palliative. In the case of preoperative values of both radically treated and palliative treated patients, we can expect an association with the actual process of carcinogenesis before treatment and so preoperative values may have a relationship to prognosis. Due to the favorable outcome of radically treated patients, survival analysis was only computed for the subgroup with palliative intervention. A relation of high preoperative plasma levels of miR-21, miR-20a, and miR-23a to shorter OS was observed. The best discrimination between patients with favorable and unfavorable outcomes was achieved by combining routinely used tumor markers with miRNAs (Figure 5). Our results show the benefit of combining currently used standard biomarkers and miRNAs for precise prognosis estimation.

The use of a combination of biomarkers reflects the current view on carcinogenesis as a multistep process that is individual in number, type, and order of qualitatively and quantitatively aberrantly expressed genes. It would be presumptive to expect that a single biomarker could cover all CRC cases. Similarly, just as there are CRC patients with low or no CEA blood serum levels, there are patients with low levels of particular oncogenic miRNA. In patients with a low preoperative level of such miRNA, we cannot expect a postoperative decrease.

Postoperative decrease is the basic criterion that must be fulfilled by markers, whose plasma levels reflect the course of the disease and could potentially be used for early detection of disease recurrence. We used these candidate molecules in the follow-up period as biomarkers for detection of recurrence. Compared to CEA and CA19-9, miRNAs displayed a higher sensitivity but lower specificity for the detection of disease recurrence. The miRNAs, as biomarkers for detection of recurrence, were compromised by a high number of false positive results. From this point of view, a combination of CEA, CA19-9, and miRNAs (miR-20a, miR-21, miR-223 and miR-23a) seems not to be beneficial. A combination would be beneficial if there were high specificities and non-overlapping low sensitivities.

In 2013, Yong et al. observed, with the use of miRNA microarray profiling and further validation with RT-qPCR, that miR-23a is elevated in both tumor tissue and blood plasma samples of CRC patients [27]. It was later published that miR-23a promotes colorectal cancer cell survival by targeting PDK4 [28], and recently, the participation of miR-23a∼27a∼24 clusters in CRC progression via reprogramming metabolism to switch from oxidative phosphorylation to glycolysis in a hypoxic tumor environment was reported [29]. The prognostic value of miR-23a has been described for many types of cancer [30], and now we have, for the first time, described a relation between the plasma levels of this miRNA and prognosis in CRC patients.

In 2010, Earle et al. revealed miR-223 to be overexpressed in CRC relative to mucosa [31]. The study of Zekri et al. in 2016 showed that miR-223 is upregulated in blood serum of CRC patients [32]. On the basis of an investigation conducted on colorectal cancer cell line SW620, Ju et al. showed that miR-223 downregulates the FoxO3a/BIM signaling pathway and so promotes proliferation [33]. Li et al. observed that patients with high miR-223 expression in CRC tumor tissue had a poor OS, and miR-223 level correlated with histology grade, metastasis, and tumor-node-metastasis (TNM) stage [34]. We described the relation of miR-223 plasma levels to prognosis.

MiR-21 is the most frequently investigated miRNA with a relation to oncologic diseases. In 2010, we described the prognostic significance of miR-21 expression in CRC tissue and detected its expression in colorectal liver metastases [14]. The diagnostic and prognostic value of tissue miR-21 levels in CRC were evaluated in a meta-analysis by Zhang et al. [35]. A recently published meta-analysis on tissue/serum miR-21 by Guraya [36] concluded that miR-21 may be used as a strong predictor for prognosis of CRC. This meta-analysis contains three studies on circulating miR-21 and four on tissue miR-21 in CRC patients. Our results on circulating miR-21 are in agreement with the conclusion of this meta-analysis.

Yang et al. found serum miR-20a to be downregulated in colorectal neoplasia patients compared to healthy controls [37]. However, Yamada et al. did not find significant differences in serum levels of miR-21 between healthy controls and early colorectal neoplasia patients [38].

As markers for the early detection of recurrence, we have selected from a large number of known miRNAs those previously identified as associated with tumor pathogenesis. For some of these miRNAs, we observed a statistically significant relationship to survival, suggesting their possible use as CRC prognostic markers. However, using these miRNAs during the follow-up period for the early detection of recurrence, we observed a low specificity. We suggest that these miRNAs also participate in other cellular processes outside tumorigenesis and could also be deregulated due to other pathological situations, e.g., the elevation of circulating miR-21 levels in cardiovascular disease has been described [39].

Therefore, increased levels of miRNA markers in the postoperative period may not be directly related to the recurrence of cancer. For this purpose, it will be necessary to look for miRNAs with exclusive involvement in tumor pathogenesis alone, which will not be easy due to the nature of the miRNA network itself. MiRNAs are involved in competing endogenous RNA crosstalk, where RNA transcripts co-regulate each other by competing for shared miRNAs, thereby titrating miRNA availability (ceRNA hypothesis) [40].

We see the main limit of interpretation of this study in the fact that while preoperative and postoperative specimens collected within a defined period of time were available in all patients, during follow-up specimens were available only in some patients, and in addition to this, they were taken at unequal time intervals. For this reason, we believe that more accurate results have been obtained when comparing preoperative with postoperative values and calculating prognosis (OS).

## 4. Materials and Methods

### 4.1. Patients

The study group consisted of 85 patients who underwent surgery for CRC at the Complex Oncology Center of the University Hospital in Pilsen between March 2008 and May 2013. The study was approved by the institutional review board and local ethics committee of the University Hospital in Pilsen. It was a retrospective study. Every patient signed an informed consent form for the use of their blood samples for the assessment of tumor markers. The clinicopathological features are summarized in Table 3.

A total of 57 patients underwent radical surgery and 28 patients a palliative intervention (either partial resection or complete resection of the primary tumor but no removal of liver metastases). Each diagnosis of CRC was verified by a pathologist and all tumors were histologically adenocarcinomas. The stage of disease was determined using the TNM system of the International Union Against Cancer (IUCC, 7th edition) [41]. The median follow-up was 3 years. The evaluation of remission and recurrence was based on response evaluation criteria in solid tumors (RECIST) [42]. The recurrence based on miRNAs (biochemical recurrence) was defined using an individual cut-off value defined as a 1.5-fold increase of the postoperative value or the presence of two consecutive plasma level values higher than the postoperative value.

### 4.2. Blood Samples

For each patient, preoperative (taken a day before surgery) and postoperative (taken during the period 5–10 days after surgery) peripheral blood samples were analyzed. Peripheral blood samples were also taken during the follow-up period (median follow-up: 27 weeks), however, not for all patients. The number of additional samples collected during the follow-up period was between 0 and 7 per patient. We evaluated 270 samples in total.

Blood samples were taken from the cubital vein. Blood plasma was prepared from 4 mL of whole blood collected into K_3_EDTA tubes by centrifugation at 1370× *g* for 10 min. The blood serum was separated by centrifugation at 1700× *g* for 10 minutes from 4 mL of blood collected in Vacuette^®^ blood collection tubes (Greiner Bio-One, Kremsmünster, Austria). The samples of plasma and serum were stored at –80 °C until use.

### 4.3. Quantitative Measurement of microRNAs

Plasma levels of the selected 22 candidate miRNAs were quantified using a reverse transcription real-time polymerase chain reaction (RT real-time PCR). Exogenous cel-miR-39 was used as a spike-in control. Total RNA (including miRNA) was extracted using miRNeasy^®^ Serum/Plasma Kit (Qiagen, Hilden, Germany) from 200 µL of blood plasma with 3.5 μL of a 1.6 × 10^8^ copies/μL working solution of cel-miR-39 (Qiagen, Hilden, Germany). A quantitative estimation of selected miRNAs (Table 4) was performed by an RT real-time PCR method using TaqMan Advanced^®^ miRNA Assays (Thermo Fisher Scientific, Foster City, CA, USA) in technical duplicates on a LightCycler^®^ 96 System (Roche, Basel, Switzerland) according to the manufacturer’s protocol (Catalog Number A25576, Publication Number 100027897, Revision C). The assays only target mature miRNAs, not their precursors.

Briefly, cDNA template preparation consisted of the following steps: a poly(A) tailing reaction, an adaptor ligation reaction, and a reverse transcription (RT) reaction using SuperScript III Reverse Transcriptase. Universal forward and reverse primers were then used in a miR-Amp reaction to increase the amount of cDNA. A total of 5 µL of diluted (1:10) cDNA template was mixed with 15 µL of PCR reaction mix containing reverse and forward primers and a TaqMan probe. The primer binding sites depended on the target miRNA sequence and the TaqMan probe annealed specifically to a complementary sequence between the forward and reverse primer sites. The PCR thermal profile was started by 1 cycle of enzyme activation at a temperature of 90 °C for 20 s followed by 40 cycles at 95 °C for 1 s and 60 °C for 20 s.

### 4.4. Processing of Real-Time PCR Data

Samples were assessed in technical duplicates. In cases where there was disagreement between results obtained from both technical duplicates, the sample assessment was repeated. To avoid plate-to-plate variation, inter-run calibrators (IRCs) were used [60]. Three samples assessed in the first run of the real-time PCR apparatus for assessment of each miRNA were used as IRCs. The samples used as IRCs were selected so that a range of obtained Ct values was covered. To obtain plasma levels of the miRNAs of interest, the ΔCt approach (2^−ΔCt^ algorithm) was used. The results are presented as relative values calculated as 2^−(Ct of miRNA of interest − Ct of normalizer)^. From the available approaches for normalization [61], exogenous reference cel-miR-39 was used as a normalizer for circulating miRNAs [21,59].

### 4.5. Quantitative Measurement of CEA and CA19-9

Serum CEA and CA19-9 levels were determined using the ACCESS CEA and ACCESS CA19-9 chemiluminescent assay with the UniCel DxI 800 Instrument (Beckman Coulter, Brea, CA, USA).

### 4.6. Statistical Analysis

Statistical analysis was performed using the software SAS 9.3 (SAS Institute Inc., Cary, NC, USA). The statistical results of the comparison of preoperative and postoperative values were calculated by the Wilcoxon signed-rank test. A *p* value of ≤0.05 was considered to be statistically significant. Prognostic significance was evaluated using the Kaplan–Meier method after finding an “optimal cut off” given by the lowest *p*-value of the logrank test for the examined markers. To evaluate the diagnostic ability of disease recurrence detection, sensitivities, specificities, positive predictive values, and negative predictive values were calculated on the basis of an individual cut-off value (defined in the Section 4.1). Receiver operating characteristic (ROC) curves were generated.

## 5. Conclusions

We observed prognostic significance of preoperative plasma levels of miR-20a, miR-23a, miR-210, and miR-223 in CRC patients. There was a decrease in plasma levels of these miRNAs after surgical removal of the tumor. The use of these miRNAs for early disease recurrence detection was affected by a low specificity in comparison with CEA and CA19-9. CEA and CA19-9 had high specificity but low sensitivity.

## Figures and Tables

**Table 1:** Differences in preoperative and postoperative levels of microRNAs, CEA, and CA19-9 according to type of surgery (radical or palliative).

Treatment	All (*n* = 85)			Radical (*n* = 57)			Palliative (*n* = 28)		
Marker	^1^Preoperative	^1^Postoperative	*p*-Value	^1^Preoperative	^1^Postoperative	*p*-Value	^1^Preoperative	^1^Postoperative	*p*-Value
**miR-20a**	0.247	0.200	0.0093	0.261	0.155	0.0004	0.246	0.266	n.s.
**miR-21**	1.050	1.007	n.s.	1.050	0.973	0.0336	1.094	1.097	n.s.
**miR-23a**	0.705	0.379	0.0013	0.964	0.559	0.0044	0.367	0.297	n.s.
**miR-29**	0.005	0.005	n.s.	0.006	0.003	n.s.	0.005	0.009	n.s.
**miR-92a**	23.264	19.698	n.s.	17.630	19.160	n.s.	38.288	26.715	n.s.
**miR-155**	0.016	0.012	n.s.	0.017	0.011	0.0375	0.013	0.014	n.s.
**miR-199a**	0.321	0.490	n.s.	0.334	0.495	0.0537	0.262	0.349	n.s.
**miR-210**	0.024	0.021	0.0392	0.021	0.013	0.0228	0.032	0.050	n.s.
**miR-223**	1.275	0.555	0.0214	1.283	0.540	n.s.	0.747	0.600	n.s.
**CEA**	3.1 ng/mL	2.4 ng/mL	<0.0001	2.0 ng/mL	1.1 ng/mL	<0.0001	18.5 ng/mL	6.8 ng/mL	0.0002
**CA19-9**	11 U/mL	9 U/mL	0.0007	8 U/mL	7 U/mL	0.0117	39 U/mL	34 U/mL	0.0278

^1^ Values shown are medians of plasma levels, microRNA as relative values. CEA—carcinoembryonic antigen.

**Table 2:** The diagnostic ability of markers in relation to the detection of disease recurrence of patients treated by radical surgery.

Marker	Recurrence *n* = 9		Remission *n* = 22		Sensitivity	Specificity	Positive Predictive Value	Negative Predictive Value
	True Positive	False Negative	True Negative	False Positive				
miR-20a	6	3	12	10	66.7%	54.6%	37.50%	80.0%
miR-21	7	2	12	10	77.8%	54.6%	41.18%	85.7%
miR-23a	6	3	13	9	66.67%	59.1%	40.00%	81.3%
miR-223	3	6	12	10	33.3%	54.6%	23.1%	66.7%
CEA	3	6	20	2	33.3%	90.9%	60.0%	76.9%
CA19-9	3	6	21	1	33.3%	95.5%	75.0%	77.8%

**Table 3:** Characteristics of patients in the study.

Variables	Number of Patients	%
**Number of Patients**	85	100
Gender		
Male	56	65.9
Female	29	34.1
**Type of Surgery**		
Radical	57	67.1
Palliative	28	32.9
**Histology**		
Adenocarcinoma	85	100
**T Stage**		
T2	10	11.8
T3	62	72.9
T4	13	15.3
**N Stage**		
N0	28	32.9
N1	40	47.1
N2	17	20.0
**M Stage**		
M0	56	65.9
M1	29	34.1
**Grade**		
1	14	16.5
2	60	70.6
3	11	12.9
**Clinical Stage**		
I	4	4.7
II	17	20.0
III	35	41.2
IV	29	34.1

**Table 4:** The analyzed microRNAs with miRBase 22 accession numbers, sequences, catalogue numbers, and references.

Symbol	miRBase 22 Accession Number	5′-3′ sequence	Thermo Fisher Scientific Cat. # A25576	References ^1^
hsa-miR-17-3p	MIMAT0000071	acugcagugaaggcacuuguag	477932_mir	[43,44]
hsa-miR-18a-5p	MIMAT0000072	uaaggugcaucuagugcagauag	478551_mir	[45,46]
hsa-miR-20a-5p	MIMAT0000075	uaaagugcuuauagugcagguag	478586_mir	[47]
hsa-miR-21-5p	MIMAT0000076	uagcuuaucagacugauguuga	477975_mir	[19,47,48,49]
hsa-miR-23a-3p	MIMAT0000078	aucacauugccagggauuucc	478532_mir	[19,50]
hsa-miR-27a-3p	MIMAT0000084	uucacaguggcuaaguuccgc	478384_mir	[19]
hsa-miR-29a-3p	MIMAT0000086	uagcaccaucugaaaucgguua	478587_mir	[43,45]
hsa-miR-92a-3p	MIMAT0000092	uauugcacuugucccggccugu	477827_mir	[46,47,51]
hsa-miR-93-5p	MIMAT0000093	caaagugcuguucgugcagguag	478210_mir	[47]
hsa-miR-96-5p	MIMAT0000095	uuuggcacuagcacauuuuugcu	478215_mir	[52]
hsa-miR-106a-5p	MIMAT0000103	aaaagugcuuacagugcagguag	478225_mir	[44,46]
hsa-miR-141-3p	MIMAT0000432	uaacacugucugguaaagaugg	478501_mir	[48,52,53]
hsa-miR-142-5p	MIMAT0000433	cauaaaguagaaagcacuacu	477911_mir	[19]
hsa-miR-143-3p	MIMAT0000435	ugagaugaagcacuguagcuc	477912_mir	[44,46]
hsa-miR-155-5p	MIMAT0000646	uuaaugcuaaucgugauagggguu	477927_mir	[54,55]
hsa-miR-183-5p	MIMAT0000261	uauggcacugguagaauucacu	477937_mir	[56]
hsa-miR-199a-3p	MIMAT0000232	acaguagucugcacauugguua	477961_mir	[57]
hsa-miR-200b-3p	MIMAT0000318	uaauacugccugguaaugauga	477963_mir	[52]
hsa-miR-200c-3p	MIMAT0000617	uaauacugccggguaaugaugga	478351_mir	[52,55]
hsa-miR-210-3p	MIMAT0000267	cugugcgugugacagcggcuga	477970_mir	[55]
hsa-miR-223-3p	MIMAT0000280	ugucaguuugucaaauacccca	477983_mir	[19,46,50,51]
hsa-miR-592	MIMAT0003260	uugugucaauaugcgaugaugu	479075_mir	[58]
cel-miR-39-3p ^2^	MIMAT0000010	ucaccggguguaaaucagcuug	478293_mir	[21,59]

^1^ Studies showing relation of particular miRNA to carcinogenesis. ^2^ Exogenous reference (spike-in control).
